# Symptom continuum reported by affective disorder patients through a structure-validated questionnaire

**DOI:** 10.1186/s12888-020-02631-y

**Published:** 2020-05-07

**Authors:** Fanjia Guo, Jingyi Cai, Yanli Jia, Jiawei Wang, Nenad Jakšić, Zsuzsanna Kövi, Marina Šagud, Wei Wang

**Affiliations:** 1 Department of Clinical Psychology and Psychiatry/ School of Public Health, Zhejiang University College of Medicine, Hangzhou, China; 2 Department of Psychiatry, University Hospital Center Zagreb, University of Zagreb School of Medicine, Zagreb, Croatia; 3 Department of General Psychology, Károli Gáspár University, Budapest, Hungary; 4 Department of Psychology, Norwegian University of Science and Technology, Trondheim, Norway

**Keywords:** Bipolar I and II disorders, Factor analyses, Major depressive disorder, Patient report, Symptom continuum

## Abstract

**Background:**

Affective disorders, such as major depressive (MDD), bipolar I (BD I) and II (BD II) disorders, are overlapped at a continuum, but their exact loci are not clear. The self-reports from patients with affective disorders might help to clarify this issue.

**Methods:**

We invited 738 healthy volunteers, 207 individuals with BD I, 265 BD II, and 192 MDD to answer a 79 item-MATRIX about on-going affective states.

**Results:**

In study 1, all 1402 participants were divided random-evenly and gender-balanced into two subsamples; one subsample was used for exploratory factor analysis, and another for confirmatory factor analysis. A structure-validated inventory with six domains of Overactivation, Psychomotor Acceleration, Distraction/ Impulsivity, Hopelessness, Retardation, and Suicide Tendency, was developed. In study 2, among the four groups, MDD scored the highest on Retardation, Hopelessness and Suicide Tendency, whereas BD I on Distraction/ Impulsivity and Overactivation.

**Conclusion:**

Our patients confirmed the affective continuum from Suicide Tendency to Overactivation, and described the different loci of MDD, BD I and BD II on this continuum.

## Background

According to current diagnostic documentation, mood disorders are largely composed of bipolar and related disorders and depressive disorders [1, 2]. Bipolar disorder has two main subtypes, i.e., I (BD I) and II (BD II). These two subtypes are different from major depressive disorder (MDD), but their clinical symptoms are mutually overlapping [3]. Therefore, they are considered to sit separately on a continuum of mood states [4], and are difficult to be separated from each other, especially between BD I and BD II. For instance, BD I is characterized by a high prevalence of reckless behavior, distractibility, psychomotor agitation, irritability and increased self-esteem [5], whereas BD II by more severe and persistent depression [6, 7] as well as more frequent and longer episodes of depression [8, 9]. Some patients, previously diagnosed as having major depressive disorders, might qualify for a bipolar disorder diagnosis [10].

In clinics, bipolar disorder usually starts with depressive episodes rather than mania or hypomania. Considering the fact that consecutive hypomanic episodes tend to be short-lived, less severe, and less socially impaired, they are difficult to differentiate from normal mood changes. Thus, bipolar disorder is often misdiagnosed as MDD [11]. Scholars also have noted that the severity of mania and depression symptoms of BD II differ from that of BD I and MDD [12]. Since hypomania has less prominent and milder symptoms, with little impact on life, work, study and interpersonal communication, it is a difficult condition to diagnose, and is easily overlooked. Indeed, the misdiagnoses can lead to ineffective treatment and increase suicide risks of affective disorder patients [13]. For example, antidepressants have little or no efficacy for depressive episodes associated with bipolar disorder [3], and might lead to phase inversion, rapid cycling state, or mixed seizures, which might worsen the condition of bipolar disorder [14]. In addition, medication or other non-pharmacologic therapies available to treat BD I and BD II are not optimally effective and their effects vary between the subtypes [15, 16].

Although the continuum hypothesis of mood symptoms fits into the clinical diagnoses of affective disorders [17], the accurate loci or segments each disorder occupies, and the mood transition or dynamic changes along of the continuum are still unclear. The accurate clinical diagnosis of an affective disorder requires trained professionals with experience and knowledge, which might be inconsistent across professionals in clinical practices, to some degree that individual description of patients might be neglected. On the other hand, symptoms reported by patients using a structure-validated symptom modeling are limited. Most symptom studies, however, were from the hospital-based medical records and professional physician interviews in clinics [3]. The self-reported questionnaire, which can be easily implemented to large samples, is an effective and straightforward method to assess many diseases and explore constructs that might be difficult to acquire through behavioral or physiological measures. Up to present, there are few studies adopting both exploratory and confirmatory factor analyses to present a structure-validated modeling to assess the symptoms of BD I, BD II and MDD, which covers all mania, hypomania and depression in a single design.

Based on previous studies [17], we hypothesize that mania, hypomania, and depression are continuous spectrum from high to low, independent at the same time, and BD I, BD II, and MDD relatively have specific components. We then developed an item-MATRIX measuring the mood symptoms according to diagnostic criteria [1, 2] and the commonly clinical-used questionnaires for depressive and bipolar-related disorders. The item examples we adopted for mania were similar to “I have much more energy than usual”, “I am much more talkative or speak faster than usual” [18], and “I need much less sleep than usual” [19]; those for hypomania were similar to “I am more self-confident”, “My thoughts jump from topic to topic” [20], “I can be exhausting or irritating for others”, and “I am more easily distracted” [19]; and those for depression were similar to “I feel worthless”, “I am making plans to commit suicide” [21], “I find it difficult to make up my mind”, and “I get tired for no reason” [22]. In order to obtain enough the item response variation, the item-MATRIX was tested in both healthy volunteers and patients with affective disorders. Two purposes of the present study were (1) to obtain a structure-validated emotional-symptom questionnaire from the item-MATRIX (Study 1), and (2) to look for the different loci or segments of emotional symptoms mostly associated with BD I, BD II and MDD through self-reports of patients (Study 2).

## Methods

### Participants

We altogether invited 1600 participants, some of whom (mostly the volunteer-controls) were excluded after a semi-structured interview or a clinical assessment (see below). Finally this investigation was carried out on 1402 participants: 738 healthy volunteers, and 207 patients with BD I, 265 BD II and 192 MDD; their demographic and grouping information are given in Table 1. The semi-structured interview was performed with each healthy participant to ensure that they were not suffering from any psychiatric or neurological problems. All patients were diagnosed by an experienced psychiatrist (WW) according to Diagnostic Criteria of American Diagnostic and Statistical Manual of Mental Disorders Fifth Edition (DSM-5) [1], were beyond their first-episode, and were confirmed to have no other psychiatric co-morbid conditions, such as schizophrenia, schizoaffective disorder, substance use disorder, eating disorder, etc. All participants were free from any drug or alcohol abuse for at least 72 h prior to the test. In addition, two coauthors were presenting onsite to aid in the proper filling of the informed consent, demographic information, and MATRIX (see below), and to guarantee corrective feedbacks. Our study design was approved by a local Ethics Committee, and all participants were informed and gave written consents to participate.

**Table 1:** Demographic and grouping information of healthy volunteers (Controls), patients with bipolar I (BD I), II (BD II) and major depressive (MDD) disorders

Characteristics	Controls	BD I	BD II	MDD
**Study 1**				
*Participant part 1 (for EFA)*				
Sample size (female)	352 (184)	111 (60)	130 (73)	108 (78)
Age (in years; mean ± SD; range)	26.27 ± 3.95 (19–42)	25.58 ± 5.75 (18–53)	27.93 ± 7.88 (18–60)	26.94 ± 8.91 (18–49)
*Participant part 2 (for CFA)*				
Sample size (female)	386 (251)	96 (50)	135 (77)	84 (49)
Age (in years; mean ± SD; range)	26.00 ± 2.75 (19–53)	26.31 ± 6.48 (18–51)	25.98 ± 8.39 (18–57)	27.15 ± 7.96 (18–53)
**Study 2**				
Sample size (female)	738 (435)	207 (110)	265 (150)	192 (127)
Age (in years; mean ± SD; range)	26.13 ± 3.39 (19–53)	25.94 ± 6.10 (18–53)	26.96 ± 8.18 (18–60)	27.03 ± 8.50 (18–53)

Note: Same participants were used in both Study 1 and Study 2, for detailed description, see text

*EFA* exploratory factor analysis, *CFA* confirmatory factor analysis

For Study 1, 1402 participants were divided into two subsamples (*n* = 701 each), there was no age (One-way ANOVA, subsample 1: F[3697] = 2.512, *p* = .06, MSE = 90.87; subsample 2: F[3.697] = 1.046, *p* = .37, MSE = 32.46) or gender (the Pearson Chi-square test with Yates’ correction, subsample 1: χ^2^ = 4.03, *p* = .26; subsample 2: χ^2^ = 3.43, *p* = .33) differences among the two subsamples. For Study 2, which were based on the participants of Study 1, there was no significant age (one-way ANOVA, F[3, 1398] = 2.45, *p* = .06, MSE = 84.81) or gender (the Pearson Chi-square test with Yates’ correction, χ^2^ = 7.52, *p* = .06) difference among the four groups either.

### The MATRIX

All participants were asked to complete the 79 MATRIX items in Chinese in a quiet room. Considering the less compliance of an individual during mania or depression, we asked participants to use simpler answer-styles of the force-choice (0 - no, 1 - yes) for some items or the Likert scale (0 - no, 1 - sometimes, 2 - most of the time) for other items, corresponding to their intensity of the on-going affective state of either depression, hypomania or mania.

### Statistical analysis

In Study 1, the answers to the 79 items from the first subsample were submitted to a principal component analysis, using SPSS Version 18.0.0 (SPSS Inc., 2009, Chicago, IL). The factor model fits were evaluated by the confirmatory factor analysis using AMOS version 17.0 (AMOS Development Corp., 2008, Crawfordville, FL) in the second subsample. The criteria for factor loadings and cross-loadings, and for selecting model fit parameters were kept consistent with a previous study [23]. When the optimal model fit was established, the factor structure of the questionnaire was developed. The internal reliability (Coefficient H) of each scale was then calculated. After both exploratory and confirmatory factor analyses, the structure-validated factor structure was formed, and a questionnaire was developed.

In Study 2, the questionnaire developed in Study 1 were tested in groups of BD I, BD II, MDD and healthy controls on one hand, using two-way ANOVA (i.e., Group (4) × Scale (6)); and also tested in groups of different mood states of mania, hypomania, bipolar depression, major depression and healthy controls on the other hand, using two-way ANOVA (i.e., Group (5) × Scale (6)). The post-hoc analysis by the Least Significant Difference test was employed to evaluate between-group differences and to estimate the effect size (Cohen’s d) for difference. A *p* value less than .05 was considered to be significant.

## Results

### Study 1: Factor structure development

**Fig. 1:**
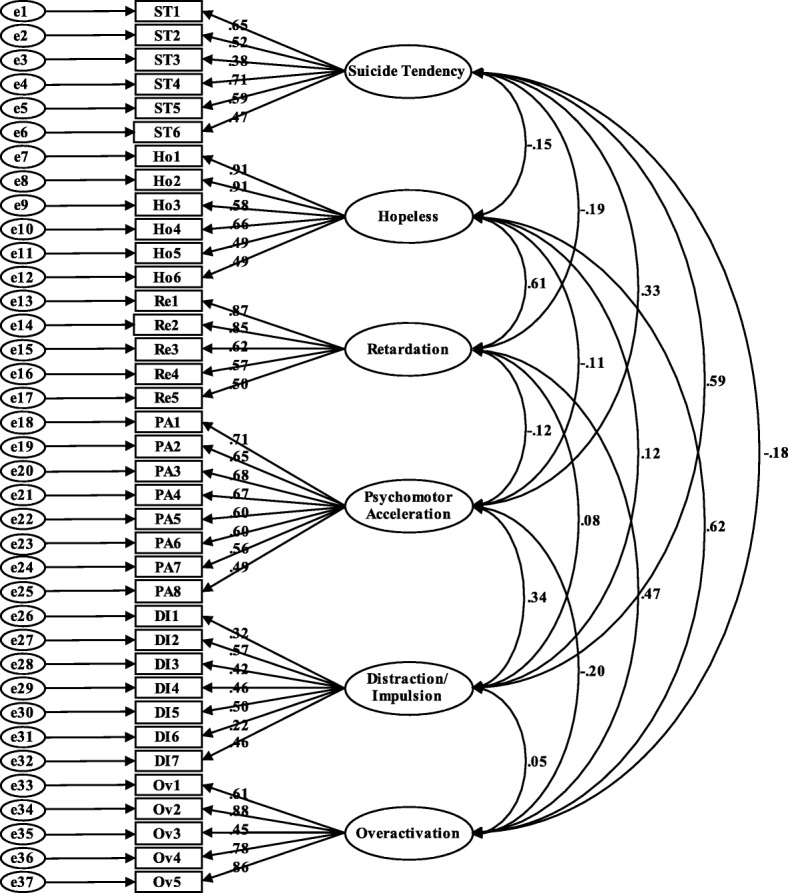
Standardized six-factor structure in 701 participants

The principal component analysis extracted 10 factors with eigenvalues larger than 1.0. The screen plot and parallel analysis results suggested a six-factor solution, and the first six factors accounted for 49.40% of the total variance. Deleting items with loadings lower than .40 or with significant cross-loadings higher than .35 on other non-target factors, we constructed a fit modeling, with 37 items which were distributed in the six factors (for the sake of brevity, loading information of all 79 items is presented as a supplementary material). In addition, the structural equation modeling (Fig. 1) confirmed that the six-factor modeling was a suitable solution (χ^2^/df = 2.86, the goodness of fit index = .88, the adjusted goodness of fit index = .86, the comparative fit index = .87, the Tucker-Lewis index = .86, the root mean square error of approximation = .052, and the standardized root mean square residual = .064).

The first factor with 8 items, e.g., “I am more self-confident”, and “My mood is higher and more optimistic”, reflects high spirits or active thoughts subjectively, thus was named “Psychomotor Acceleration” (internal reliability of .88). The second factor with 6 items, e.g., “I feel sad” and “I find it difficult to make up my mind”, narrates the characteristics of depression, including sorrow, self-accusation, hesitation, etc., “Hopelessness” (internal reliability, .85). The third factor with 5 items, e.g., “I think about committing suicide”, and “I am making plans to commit suicide”, describes the thoughts and behavior of suicide, “Suicide Tendency” (internal reliability, .87). The fourth factor with 6 items, e.g., “I have much more energy than usual” and “I am much more talkative or speak faster than usual”, describes the characteristics of increase of targeted behaviors and speaking, agitated mental activity, and tendency to high-risk activities, “Overactivity” (internal reliability, .81). The fifth factor with 6 items, e.g., “I speak less”, and “The speed at which I do things is lower”, represents lack of motivation for speaking and acting, “Retardation” (internal reliability, .84). The six factor with 7 items, e.g., “I am more easily distracted” and “I take more risks in my daily life”, is a mixture of distraction, anxiety and impulsion descriptions, “Distraction/ Impulsivity” (internal reliability, .74) (Table 2).

**Table 2:** Factor loadings on the six factors in 701 participants

Factor	Items	Factor					
		1	2	3	4	5	6
Psychomotor Acceleration	I am more self-confident	**.76**	−.06	−.03	.08	−.01	−.09
	My mood is higher and more optimistic	**.75**	.00	−.08	.04	−.03	−.01
	I do think more quickly or more easily or both	**.73**	.06	−.05	−.02	−.06	−.06
	I think faster	**.67**	.09	−.01	.06	.03	.11
	I enjoy my work more	**.65**	−.08	−.04	.11	−.08	−.09
	I am less shy or inhibited	**.65**	.02	−.02	.10	.11	.15
	I plan more activities or projects	**.59**	−.04	−.08	.07	−.07	.16
	I am physically more active (in sports etc.)	**.56**	−.11	−.01	.07	.01	.23
Hopelessness	I feel depressed	−.04	**.77**	.19	−.05	.28	.01
	I feel sad	−.04	**.77**	.21	−.05	.31	.02
	I find it difficult to make up my mind	−.01	**.68**	.27	−.05	.17	.07
	I feel worthless	−.09	**.67**	.13	−.07	.24	.08
	I am tired	.04	**.59**	−.01	−.06	.03	.08
	I complain	.02	**.58**	.02	.18	.18	.02
Suicide Tendency	I think about committing suicide	−.05	.22	**.82**	−.01	.13	.03
	I am making plans to commit suicide	−.04	−.03	**.78**	−.03	.08	−.01
	I think I would be better off dead	−.11	.34	**.73**	.02	.11	.05
	I think about death	−.04	.29	**.69**	.02	.18	.04
	I am making a suicide attempt	−.05	.01	**.69**	.01	.11	.10
Overactivation	I have much more energy than usual	.15	−.21	−.10	**.72**	−.08	−.04
	I am much more talkative or speak faster than usual	.09	.07	−.02	**.68**	−.12	.17
	I am much more interested in sex than usual	.11	−.08	−.01	**.67**	.04	.11
	I am much more social or outgoing than usual, for example, I telephone friends in the middle of the night	.11	−.04	−.04	**.62**	.03	.14
	I sleep much less than usual and find I do not really miss it	.07	.08	.06	**.59**	.05	.03
	I feel so good or so hyper that other people think I am not my normal self, or I am so hyper that I get into trouble?	−.06	.03	.07	**.49**	−.05	.27
Retardation	I speak less	.00	.24	.09	−.07	**.82**	−.02
	My urge to speak is less	.00	.27	.13	−.07	**.80**	.03
	The speed at which I do things is lower	−.08	.32	.19	−.06	**.57**	.02
	My interest in sex is less	−.03	.21	.14	−.07	**.50**	.03
	My appetite is less	.02	.09	.07	.14	**.47**	.18
Distraction/ Impulsion	I am more easily distracted	−.21	.09	−.05	.02	.02	**.61**
	I take more risks in my daily life (in my work and/or other activities)	.19	.13	.00	.09	.11	**.58**
	My thoughts jump from topic to topic	.27	−.18	.08	.16	.14	**.57**
	I spend more or too much money	.20	.07	.06	.02	−.03	**.54**
	I tend to drive faster or take more risks when driving	.02	.01	.03	.17	.08	**.53**
	I can be exhausting or irritating for others	−.24	.15	.06	.17	−.13	**.47**
	I am more flirtatious, or am sexually more active, or both	.30	−.02	.07	.17	.10	**.41**

Loadings higher than .40 are presented in bold for clarity

### Study 2: Factor structure application

The mean scores of the six factors were significantly different between four groups of BD I, BD II, MDD and healthy controls (main effect, F[3,1398] = 498.74, *p* < .001, MSE = 1618.52; scale effect, F[5,1396] = 1026.05, *p* < .001, MSE = 3329.78; group × scale interaction effect, F[15,1386] = 176.76, *p* < .001, MSE = 573.63). There were significant between-group differences between the four groups on all factors (Psychomotor Acceleration: F[3,1398] = 150.11, *p* < .001, MSE = 636.61; Hopelessness: F[3,1398] = 338.86, *p* < .001, MSE = 1867.08; Suicide Tendency: F[3,1398] = 141.46, *p* < .001, MSE = 255.36; Overactivity: F[3,1398] = 345.36, *p* < .001, MSE = 581.15; Retardation: F[3,1398] = 193.39, *p* < .001, MSE = 670.38; Distraction/ Impulsivity: F[3,1398] = 163.78, *p* < .001, MSE = 373.41). Post-hoc tests showed that the scale scores of Hopelessness, Suicide Tendency and Retardation were significantly higher in MDD than other groups, followed by BD II; BD I showed highest scores on Overactivation and Distraction/ Impulsivity. Both BD I and BD II presented high scores on Psychomotor Acceleration, while BD II and MDD showed low scores in Overactivation (Table 3).

**Table 3:** Scale scores (mean ± S.D.) of the six factors in healthy volunteers (controls, *n* = 738), bipolar I (*n* = 207), bipolar II (*n* = 265) and major depressive (*n* = 192) disorder patients

Factor	Controls	Bipolar I	Bipolar II	Major depressive	Cohen’s d
Psychomotor Acceleration	5.69 ± 2.28	7.21 ± 1.36^a^	7.28 ± 1.08^a^	3.59 ± 2.72^abc^	.719 ~ 1.962
Hopelessness	1.92 ± 1.93	2.89 ± 2.25^a^	5.46 ± 3.15^ab^	7.22 ± 2.61^abc^	.484 ~ 2.538
Suicide Tendency	.15 ± .58	.43 ± 1.08^a^	1.23 ± 1.98^ab^	2.24 ± 2.28^abc^	.389 ~ 1.807
Overactivation	1.19 ± 1.42	4.24 ± 1.28^a^	1.03 ± 1.12^b^	.90 ± 1.04^ab^	.215 ~ 2.853
Retardation	1.22 ± 1.42	1.77 ± 1.91	3.55 ± 2.44^ab^	4.22 ± 2.32^abc^	.280 ~ 1.823
Distraction/ Impulsivity	1.85 ± 1.54	4.33 ± 1.57^a^	3.04 ± 1.44^ab^	2.09 ± 1.40^bc^	.667 ~ 1.604

Note: ^a^*p* < .01 vs. controls; ^b^*p* < .01 vs. Bipolar I; and ^c^*p* < .01 vs. Bipolar II

When different affective states were considered, the mean scores of the six factors were significantly different between groups of mania, hypomania, bipolar depression, MDD and healthy controls (main effect, F[4,1397] = 352.05, *p* < .001, MSE = 1215.40; scale effect, F[5,1396] = 593.03, *p* < .001, MSE = 2047.37; group × scale interaction effect, F[20,1381] = 99.67, *p* < .001, MSE = 344.09). There were significant between-group differences among different groups on all factors (Psychomotor Acceleration: F[4,1397] = 112.53, *p* < .001, MSE = 477.56; Hopelessness: F[4,1397] = 219.66, *p* < .001, MSE = 1284.18; Suicide Tendency: F[4,1397] = 99.69, *p* < .001, MSE = 182.63; Overactivity: F[4,1397] = 95.29, *p* < .001, MSE = 219.49; Retardation: F[4,1397] = 124.79, *p* < .001, MSE = 531.88. Distraction/ Impulsivity: F[4,1397] = 107.84, *p* < .001, MSE = 240.10). Post-hoc tests also showed that patients during mania state scored higher on overactivity, while patients during depression scored higher on Hopelessness and Suicidal Tendency (Table 4).

**Table 4:** Scale scores (mean ± S.D.) of the six factors in healthy volunteers (controls, *n* = 738), patients in manic (*n* = 45), hypomanic (*n* = 111), bipolar depressive (*n* = 316), and major depressive (*n* = 192) states

Factor	Controls	Manic state	Hypomanic state	Bipolar depressive	Major depressive	Cohen’s d
Psychomotor Acceleration	5.69 ± 2.28	7.33 ± 1.26^a^	7.30 ± .96^a^	7.22 ± 1.28^a^	3.59 ± 2.72^abcd^	.734 ~ 1.859
Hopelessness	1.92 ± 1.93	2.44 ± 2.26	5.45 ± 2.87^ab^	4.21 ± 3.08^abc^	7.22 ± 2.61^abcd^	.410 ~ 2.538
Suicide Tendency	.15 ± .58	.38 ± .98	1.35 ± 2.07^ab^	.78 ± 1.60^ac^	2.24 ± 2.28^abcd^	.329 ~ 1.807
Overactivation	1.19 ± 1.42	4.09 ± 1.49^a^	1.13 ± 1.15^b^	2.66 ± 2.03^abc^	.90 ± 1.02^b^	.725 ~ 2.803
Retardation	1.67 ± 1.64	2.13 ± 1.80	3.85 ± 2.52^ab^	3.37 ± 2.60^ab^	5.04 ± 2.28^abcd^	.493 ~ 1.882
Distraction/ Impulsivity	1.80 ± 1.49	3.80 ± 1.78^a^	2.95 ± 1.37^ab^	3.69 ± 1.59 ^ac^	1.89 ± 1.30^bc^	.482 ~ 1.362

Note: ^a^*p* < .01 vs. controls; ^b^*p* < .01 vs. manic; ^c^*p* < .01 vs. hypomanic; and ^d^*p* < .01 vs. bipolar depressive

## Discussion

**Fig. 2:**
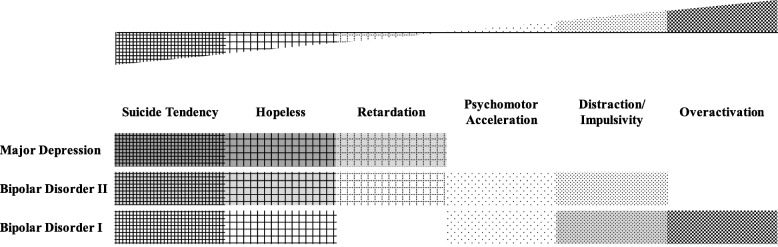
Locations of affective symptom of major depressive, bipolar I and II disorders along a continuum

After both exploratory and confirmatory factor analyses on self-reports, we have developed a fit modeling, with 37 items which were distributed in the six factors (Study 1). These factors were located in a continuum from high to low end of the emotional states: Overactivation, Psychomotor Acceleration, Distraction/ Impulsivity, Hopelessness, Retardation, and Suicide Tendency, which supports our first hypothesis. Keeping up with our second hypothesis, patients with BD I, BD II and MDD described themselves differently when referring to the loci or segments of the continuum they occupied (Study 2, Fig. 2), which were supported by results from different affective states as well.

The first factor, Psychomotor Acceleration, refers to the increased intensity of emotion which is often used to characterize mania and hypomania, as described in hypomanic state previously [9]. Both BD I and BD II patients exhibited similar emotional symptoms, such as psychomotor agitation and strong self-esteem [9, 24]. The fourth factor, Overactivation, describes manic and hypomanic thoughts and behaviors, as previously described in manic state [25]. BD I, instead of BD II, scored highest among all the four groups, showing that BD I patients have more prominent agitated and excited states [5, 26]. MDD patients, however, scored lowest on both Psychomotor Acceleration and Overactivation [3]. One group of scholars also have suggested that, apart from psychotic symptoms, manic and hypomanic episodes have the same symptom profile and differ only in the degree of severity. Studies have reported that manic episodes exhibit a broader spectrum of symptoms [26]. In addition, many scholars have found a prominent mood characteristic to be “elated mood” in BD II compared to similar rates of “elated mood” and “irritable mood” in BD I [9].

The second factor, Hopelessness, describes the loss of incentives, low self-worth, and sad emotions, in thoughts (cognition), as previously described in depression [27, 28]. In terms of scores of the Hopelessness, MDD, BD II, and BD I had diminished scores, which were significantly different from each other, with MDD the lowest. The results were in accordance with previous results in these patients [29, 30]. The fifth factor, Retardation, refers to slow-down behavior, amotivation, in daily activities, also as previously described in depression [31]. It describes the features of lack of motivation, as previously reported in depressive state [32, 33], found to be prominent in our MDD and BD II. Former studies have declared that BD II patients experienced major depressive episodes more frequently than those with BD I [34]. Sub-syndromal depressive episodes, which do not fully meet the criteria for major depressive episode but may contribute to a decline in occupational and social outcome, have also been reported more in BD II [35–37].

The third factor, Suicide Tendency, describes the thoughts and plans to commit suicide which has been reported widely in depressive state [38–40]. In our Study 2, MDD scored highest on this domain, followed by BD II and BD I. Previous studies have shown that the disability rates of major depressive and bipolar disorder patients ranked first and second, respectively, around the world [41]. The reasons were mostly related to suicide ideation and behavior, which often occurred in severe depression state or mixed states [42], often found in BD II [9, 43, 44]. Our findings on the highest suicide ideation scores in MDD group might be attributed to the fact that, all MDD patients were currently depressed, only 60% BD II and 80% BD I patients, were in depressed phase.

The sixth factor, Distraction/Impulsivity, describes the less-calmed or dysfunctional attention states, as previously documented in affective disorders [30, 45, 46]. For instance, a 2-year follow-up study has shown that attention is one of the cognitive domains that are persistently affected in bipolar disorder patients, and the deficits seem stable and are not worse over time [47]. The inattention symptom was predictive of change in depression severity over the course of treatment and overall treatment outcome as well [48]. On the other hand, depressive disorder [45, 49, 50] and bipolar disorder patients have displayed high levels of impulsivity [51, 52] and aggressiveness [53] no matter during manic episodes, depressive episodes, or remission stages, and the impulsivity level has been lowered in bipolar disorder patients after efficient treatments [54].

However, there were at least three design limitations of our current investigation. Firstly, we excluded items measuring substance-abuse and delusion, which are the two main features of mania, and other disease-controls such as substance misuse disorder or schizophrenia would be useful additions. Secondly, we recorded neither normal nor disordered personality traits in our participants, as they might also be related to emotional states. Thirdly, our design was cross-sectional, which addressed only the concurrent affective states of each participant. A longitudinal design of emotional measurement, especially in BD I and BD II, and from their first episode on, would be more informative to construct a dynamic model of affective disorders, and distinguish state vs. trait features.

## Conclusions

Through self-reports, we have demonstrated a structure-validated measure of emotional state, with 6 domains (37 items) in a continuum, from high to low end, namely Overactivation, Psychomotor Acceleration, Distraction/ Impulsivity, Hopelessness, Retardation, and Suicide Tendency. Patients with BD I, BD II and MDD scored differently from each other along the continuum (Fig. 2). Although our study has not provided the pictures of affective-state transition within the affective disorders, it has demonstrated the existence of and relationship between individual affective disorders.

## Supplementary information

**Additional file 1: Table S1.** Factor loadings on the six factors of 79 items.

## Data Availability

All data generated or analyzed during this study are available from the corresponding author on reasonable request.

## Abbreviations

MDD: Major depressive disorder
BD I: Bipolar I disorder
BD II: Bipolar II disorder
DSM: Diagnostic Criteria of American Diagnostic and Statistical Manual of Mental Disorders
SPSS: Predictive analytics software statistics
AMOS: Analysis of moment structures
