# Supplementary material for: Symptom continuum reported by affective disorder patients through a structure-validated questionnaire
Source: BMC Psychiatry. 2020 May 7;20:207. doi: 10.1186/s12888-020-02631-y (PMC7206809; doi:10.1186/s12888-020-02631-y)
Supplement: Supplementary file 1 — Additional file 1: Table S1. Factor loadings on the six factors of 79 items. [file 12888_2020_2631_MOESM1_ESM.docx]

**Table S1**. Factor loadings on the six factors of 79 items.

| Items | Factor | | | | | |
| --- | --- | --- | --- | --- | --- | --- |
|  | 1 | 2 | 3 | 4 | 5 | 6 |
| I am more confident | **.68** | -.07 | -.04 | .08 | -.23 | -.05 |
| I have more ideas, I am more creative | **.66** | -.03 | .04 | .07 | -.03 | .01 |
| My mood is higher, more optimistic | **.66** | -.01 | -.04 | .03 | -.25 | -.10 |
| I think faster | **.65** | .06 | .02 | .08 | -.08 | -.04 |
| I feel more energetic and more active | **.64** | -.06 | -.05 | .11 | -.28 | -.06 |
| I am less shy or inhibited | **.64** | -.06 | .04 | .07 | .00 | -.02 |
| I do think more quickly and/or more easily | **.64** | .00 | -.01 | .01 | -.21 | -.02 |
| I am more sociable (make more phone calls, go out more) | **.62** | .05 | .00 | .07 | .09 | .01 |
| I engage in lots of new things | **.61** | -.03 | -.06 | .07 | .06 | -.04 |
| I plan more activities or projects | **.60** | -.03 | -.02 | .07 | -.06 | -.06 |
| I want to meet or actually do meet more people | **.59** | .02 | .01 | .10 | .13 | .00 |
| I make more jokes or puns when I am talking | **.56** | .08 | .01 | .04 | .08 | -.04 |
| I am physically more active (sport, etc.) | **.56** | -.07 | -.01 | .08 | -.03 | .00 |
| I enjoy my work more | **.56** | -.05 | -.06 | .11 | -.24 | .00 |
| I talk more | **.55** | .10 | -.01 | .10 | .02 | -.03 |
| I am more interested in sex, and/or have increased sexual desire | **.44** | .03 | .06 | .06 | .26 | .05 |
| I am more flirtatious and/or am sexually more active | **.43** | -.01 | .05 | .14 | .33 | .05 |
| I take more risks in my daily life (in my work and/or other activities) | **.40** | -.03 | -.02 | .17 | .38 | .02 |
| I want to travel and do travel more | .35 | .09 | .03 | .10 | .16 | .01 |
| I wear more colourful and more extravagant clothes/make-up | .34 | .01 | .01 | .06 | .24 | .04 |
| My thoughts jump from topic to topic | .33 | .23 | .04 | .16 | .31 | -.05 |
| I need less sleep | .25 | -.09 | .10 | .24 | .12 | .03 |
| I feel worthless | -.03 | **.71** | .23 | -.05 | .02 | .21 |
| I find it difficult to make up my mind | -.03 | **.70** | .25 | -.02 | .06 | .09 |
| I am distracted | .00 | **.69** | .31 | -.04 | .05 | .18 |
| I have trouble making decisions | -.03 | **.68** | .24 | .00 | .02 | .10 |
| I feel sad | -.03 | **.68** | .34 | -.09 | .05 | .24 |
| I have trouble concentrating | .03 | **.67** | .32 | -.04 | -.01 | .10 |
| I feel depressed | -.03 | **.67** | .35 | -.08 | .06 | .23 |
| I feel guilty | .08 | **.55** | .12 | -.03 | .04 | .24 |
| I am tired | .01 | **.49** | .29 | .01 | -.04 | -.03 |
| I feel more “blue” in the morning than later in the day | .00 | **.45** | .31 | -.07 | .09 | .16 |
| I am so easily distracted by things around me that I have trouble concentrating or staying on track | .10 | **.40** | .11 | .30 | .12 | -.08 |
| I complain | .07 | .36 | .14 | .00 | .08 | .35 |
| I speak less | .00 | .26 | **.62** | -.14 | .01 | .10 |
| My urge to speak is less | .03 | .29 | **.61** | -.12 | .02 | .12 |
| The speed at which I do things is lower | -.08 | .35 | **.61** | -.10 | .07 | .07 |
| My interest in doing things is lower | -.03 | .44 | **.59** | -.18 | .05 | .13 |
| I have slept less | .02 | .15 | **.58** | .06 | .08 | .04 |
| The speed of my thinking seems to be lower | .00 | .36 | **.56** | -.11 | .06 | .04 |
| My appetite is less | .04 | .03 | **.55** | .15 | .09 | .12 |
| My energy level is lower | .06 | .33 | **.55** | -.07 | .02 | .02 |
| The amount of satisfaction I get out of doing things is less | -.01 | .43 | **.53** | -.09 | .07 | .08 |
| I have wakened in the middle or the night more | -.02 | .07 | **.53** | .06 | .15 | .07 |
| I have gotten up earlier than usual more | .07 | .07 | **.53** | .14 | .01 | .02 |
| I am restless | .03 | .44 | **.53** | .03 | .02 | .09 |
| My ability to think rationally is lower | -.03 | .38 | **.52** | -.08 | .09 | .06 |
| My weight is losing | -.08 | -.01 | **.50** | .10 | .12 | .12 |
| My interest in sex is less | -.04 | .18 | **.50** | -.10 | .10 | .14 |
| I complain | .02 | .33 | **.48** | .10 | .01 | .07 |
| I am irritable | .00 | .27 | **.45** | .14 | .06 | .14 |
| I have much more energy than usual | .14 | -.20 | -.10 | **.75** | -.10 | -.06 |
| I am more active of do many more things than usual | .16 | -.19 | -.08 | **.73** | -.07 | -.07 |
| I feel much more self-confident than usual | .17 | -.20 | -.12 | **.69** | -.06 | -.05 |
| I am much more talkative or speak faster than usual | .15 | .05 | -.06 | **.68** | .09 | -.06 |
| I am much more interested in sex than usual | .17 | -.06 | -.03 | **.54** | .15 | .00 |
| I am much more social or outgoing than usual, for example, I telephone friends in the middle of the night | .20 | -.04 | -.01 | **.54** | .13 | .00 |
| I sleep much less than usual and find I do not really miss it | .09 | -.03 | .17 | **.50** | .04 | .05 |
| I feel so good or so hyper that other people think I am not my normal self, or I am so hyper that I get into trouble | .07 | .06 | .00 | **.50** | .25 | .00 |
| I do things that are unusual for me or that other people may have thought are excessive, foolish, or risky | .13 | .19 | .05 | **.46** | .27 | .10 |
| Thoughts race through my head or I can’t slow my mind down | .10 | .29 | .14 | **.43** | .16 | .05 |
| I am so irritable that I shout at people or started fights or arguments | .04 | .26 | .15 | .27 | .25 | .13 |
| I get into more quarrels | -.10 | .05 | .09 | .10 | **.58** | .07 |
| I can be exhausting or irritating for others | -.17 | .13 | .07 | .20 | **.56** | .01 |
| I am more impatient and/or get irritable more easily | -.19 | .13 | .01 | .21 | **.51** | .00 |
| I drink more alcohol | .06 | .02 | .08 | -.07 | **.49** | .07 |
| I smoke more cigarettes | -.05 | .00 | .07 | -.08 | **.48** | .03 |
| I am more easily distracted | -.07 | .17 | -.02 | .22 | **.44** | -.08 |
| I take more drugs | -.09 | -.08 | .11 | -.04 | **.43** | .12 |
| I tend to drive faster or take more risks when driving | .17 | .03 | .06 | .15 | **.40** | .02 |
| I spend more/too much money | .31 | .13 | -.03 | .10 | .35 | .02 |
| Spending money gets me or my family into trouble | .06 | .09 | .05 | .21 | .29 | .11 |
| I drink more coffee | .06 | -.12 | .13 | .01 | .27 | .03 |
| I think about committing suicide | -.06 | .28 | .21 | -.05 | .03 | **.77** |
| I make plans to commit suicide | -.09 | .07 | .11 | .00 | .08 | **.74** |
| I think I would be better off dead | -.09 | .37 | .20 | -.03 | .08 | **.70** |
| I think about death | -.05 | .32 | .25 | -.02 | -.01 | **.68** |
| I make a suicide attempt | -.05 | .08 | .14 | .01 | .11 | **.62** |
| I think about relatives or friends who have dead | .00 | .23 | .12 | .05 | .10 | .34 |

Loadings higher than .40 are presented in bold for clarity. For names of latent factors, see Table 2 in text.
